# How do spatiotemporal interactions drive the non-grain conversion of cultivated land? A multi-model study from Henan Province, China

**DOI:** 10.1038/s41598-026-51000-4

**Published:** 2026-04-29

**Authors:** Yifei Zheng, Yingchao Li, Xiaotong Xie

**Affiliations:** 1 China University of Geosciences (Wuhan), Wuhan, 430074 China; 2 Henan Agricultural University, Zhengzhou, 450002 China

**Keywords:** Non-grain production of cultivated land, Spatiotemporal analysis, Spatial autocorrelation, Geographical detector, Driving factors, Environmental social sciences, Geography, Geography

## Abstract

China faces the perennial challenge of ensuring food security due to its vast population and limited arable land. Against this backdrop, curbing uncontrolled non-grain use of cultivated land has become a critical policy concern. Although existing studies have examined the spatiotemporal patterns of non-grain conversion, the intrinsic temporal–spatial linkages and interactive driving mechanisms remain poorly understood. To address this gap, this study conducts a county-level analysis in Henan Province from 2012 to 2022, integrating spatiotemporal variance analysis, spatial autocorrelation modeling, and geographical detector techniques. The findings reveal that: (1) the non-grain conversion rate followed a U-shaped trend, declining initially before rising again, with marked regional disparities and pronounced clustering in the southwest and central regions; (2) temporal factors outweighed spatial heterogeneity in driving changes, with socioeconomic variables—particularly population density—emerging as the dominant influence; and (3) the interaction between population density and GDP exhibited the strongest explanatory power, underscoring the compounding effects of socioeconomic drivers. Based on these insights, the study proposes tailored policy measures to mitigate excessive non-grain expansion and safeguard sustainable grain production. It also contributes a novel spatiotemporal–mechanism analytical framework applicable to similar regional contexts.

## Introduction

### Background

Grain security is a fundamental national priority and a cornerstone of social stability and sustainable development. China, with approximately 9% of the world’s arable land, feeds nearly 20% of the global population, yet faces the persistent challenge of supporting a large population with limited farmland. In this context, maintaining stable grain production capacity, safeguarding the red line of cultivated land, and strictly enforcing the protection system for Permanent Basic Farmland have become essential strategies for ensuring national food security. In recent years, rapid industrialization and urbanization have intensified pressures on agricultural production. The low comparative profitability of grain farming, coupled with rising agricultural input costs, has driven many farmers to shift toward high-value cash crops in pursuit of higher returns. At the same time, land conversion for construction and ongoing agricultural restructuring have accelerated the non-grain use of cultivated land. These developments have led to imbalances in the agricultural sector, ecological degradation, and weakened resilience in rural systems, collectively posing a serious threat to China’s grain security and long-term food stability^1,2^. According to the China Statistical Yearbook 2023, although the total sown area of crops increased in 2022 compared to the previous year, the sown areas of three major grain crops—rice, wheat, and corn—declined by 908,000 hectares, 49,000 hectares, and 254,000 hectares, respectively. This trend underscores how the non-grain conversion of cultivated land has emerged as a major risk to food security. Excessive non-grain use not only threatens grain supply but also disrupts agricultural production structures, impairs ecosystem functions, and undermines the sustainability of rural economies. In response, the Chinese government has introduced a range of policies to reinforce the emphasis on grain production and curb the uncontrolled expansion of non-grain farming. The 2023 Central Policy Document outlined key measures to strengthen grain output and stabilize agricultural supply, highlighting initiatives such as revitalizing the seed industry and enhancing production capacity. The 2024 Central Policy Document further emphasized the need to secure the output of grain and major agricultural products, stabilize sown area, raise per-unit yields, and maintain annual grain production above 650 million metric tons. Importantly, it explicitly called for resolutely preventing the "non-agriculturalization" and "non-grain conversion" of cultivated land and for strictly implementing the special protection system for Permanent Basic Farmland.

Henan Province serves as a cornerstone of China’s agricultural system, accounting for 10% of the nation’s total grain output and 25% of its wheat production, thus playing a pivotal role in safeguarding national food security. However, under mounting pressures from a dense population, limited land resources, and ongoing economic transformation, the province’s cultivated land area has continued to shrink. According to the 2022 National Land Survey, Henan’s cultivated land decreased by 59.81 thousand hectares since 2017, with only 7.51 million hectares remaining—a situation that poses serious challenges to the enforcement of permanent basic farmland protection policies. Against this backdrop, examining the spatiotemporal evolution and driving mechanisms of non-grain conversion in Henan Province is not only a regional concern but also a matter of national strategic importance. Due to its unique socioeconomic development trajectory and natural conditions, Henan exhibits distinct patterns and drivers of non-grain conversion compared to other Chinese provinces. Biophysically, Henan features a pronounced transitional topography from western mountains to eastern plains, creating distinct agricultural constraints. Socioeconomically, as a core grain-producing region with nearly 100 million people, it faces acute human-land tension under stringent national food security mandates. Unlike coastal areas facing industrial encroachment or western regions experiencing farmland abandonment, Henan’s primary land-use conflict is internal agricultural restructuring. Driven by the profit gap between staple grains and cash crops, smallholder farmers are increasingly adjusting their planting structures. This unique convergence of severe demographic pressure, strict policies, and topographical diversity drives its distinct non-grain conversion pattern. Nevertheless, research on non-grain conversion at the county level in Henan remains limited. To address this gap, this study adopts county-level units in Henan as the analytical scope and employs spatiotemporal variation analysis, spatial autocorrelation modeling, and geodetector methods to systematically examine the evolution and drivers of non-grain conversion of cultivated land from 2012 to 2022. By investigating the responsiveness of non-grain conversion to various factors and their interactions, this research enhances the mechanistic understanding of cultivated land use change and provides a scientific basis for implementing the national grain security strategy, strengthening the protection of Permanent Basic Farmland, and optimizing land-use planning.

### Literature review

Currently, there is no universally established definition of cultivated land non-grain conversion in the international academic literature. Research outside China has primarily focused on the socioeconomic and ecological consequences of rapid cash crop expansion, with increasing attention being paid to crop diversity and sustainable agricultural transitions. Such studies often aim to shift away from monoculture systems by promoting climate-resilient farming practices that mitigate environmental stressors and enhance sustainability^3,4^. Methodologically, international scholars commonly employ remote sensing techniques to monitor agricultural land-use changes. These are often supplemented by macroeconomic and social statistics, integrated with linear regression models and spatial analysis, to identify conversion patterns and project future land-use trajectories^5^. In terms of influencing factors, existing research highlights that cultivated land conversion stems from the interaction of multiple drivers, where cross-factor synergies substantially increase the complexity of land-use governance^6^.

In China, the quantification of non-grain conversion of cultivated land relies on several methodological approaches, including the grain cultivation ratio^7^, the proportion of non-grain sown area to total crop sown area^8^, and the ratio of non-grain cultivated land transfer area to total agricultural land transfer area^9^. Existing research in the Chinese context has primarily focused on analyzing the causes, identifying spatial patterns, and interpreting the underlying mechanisms of non-grain conversion. Studies have been conducted at multiple scales, ranging from the national^10^ and provincial^11^ levels to county-level^12^ and agricultural economic zones^13^. Methodologically, Chinese scholars often integrate geospatial technologies with spatial econometric models to quantify the extent of non-grain conversion^14,15^. Analytical techniques such as principal component analysis, geodetector models, and geographically weighted regression are widely employed, with a predominant focus on household-level and socioeconomic drivers. These studies examine the influence of factors such as farmer income, industrial structure, and agricultural mechanization on non-grain conversion dynamics^16,17^. Notably, researchers including Song et al.^18^ and Liu et al.^19^ have highlighted that even moderate farmland transfer can intensify non-grain utilization. Additional studies have explored the relationship between non-grain conversion and land quality^20^ or topographic conditions^21^, further enriching the understanding of its multidimensional drivers.

While research on non-grain conversion of cultivated land has achieved considerable maturity, several critical limitations persist in specific domains. Most existing methodologies rely predominantly on temporal grid data comparisons to investigate spatiotemporal evolution patterns, often neglecting intrinsic spatiotemporal correlations and dynamic interactions. This oversight results in an oversimplification of the inherently complex conversion processes. Regarding driving mechanisms, prevailing studies tend to focus excessively on single-scale, macro-level determinants while lacking systematic examination of interactive effects among drivers. This narrow focus hinders a comprehensive understanding of the multifactorial compound mechanisms underlying non-grain conversion and leaves the interplay between natural and anthropogenic factors underexplored. To address these gaps, this study develops an integrated analytical framework that incorporates interactivity, dynamics, and uncertainty in spatiotemporal variations. Our methodology systematically examines non-grain conversion through three complementary approaches: spatiotemporal variation modeling to characterize dynamic patterns, spatial autocorrelation analysis to identify aggregation dynamics across temporal phases, and geodetector modeling to disentangle natural versus anthropogenic driving forces and their interactions across temporal and spatial dimensions. This multidimensional approach—encompassing temporal, spatial, natural, and anthropogenic perspectives—enables a more holistic and scientifically grounded understanding of non-grain conversion phenomena. The findings provide a comprehensive evidence base for formulating targeted policies to curb uncontrolled agricultural land transitions and promote sustainable land-use management.

## Materials and methods

### Study area overview

**Fig. 1:**
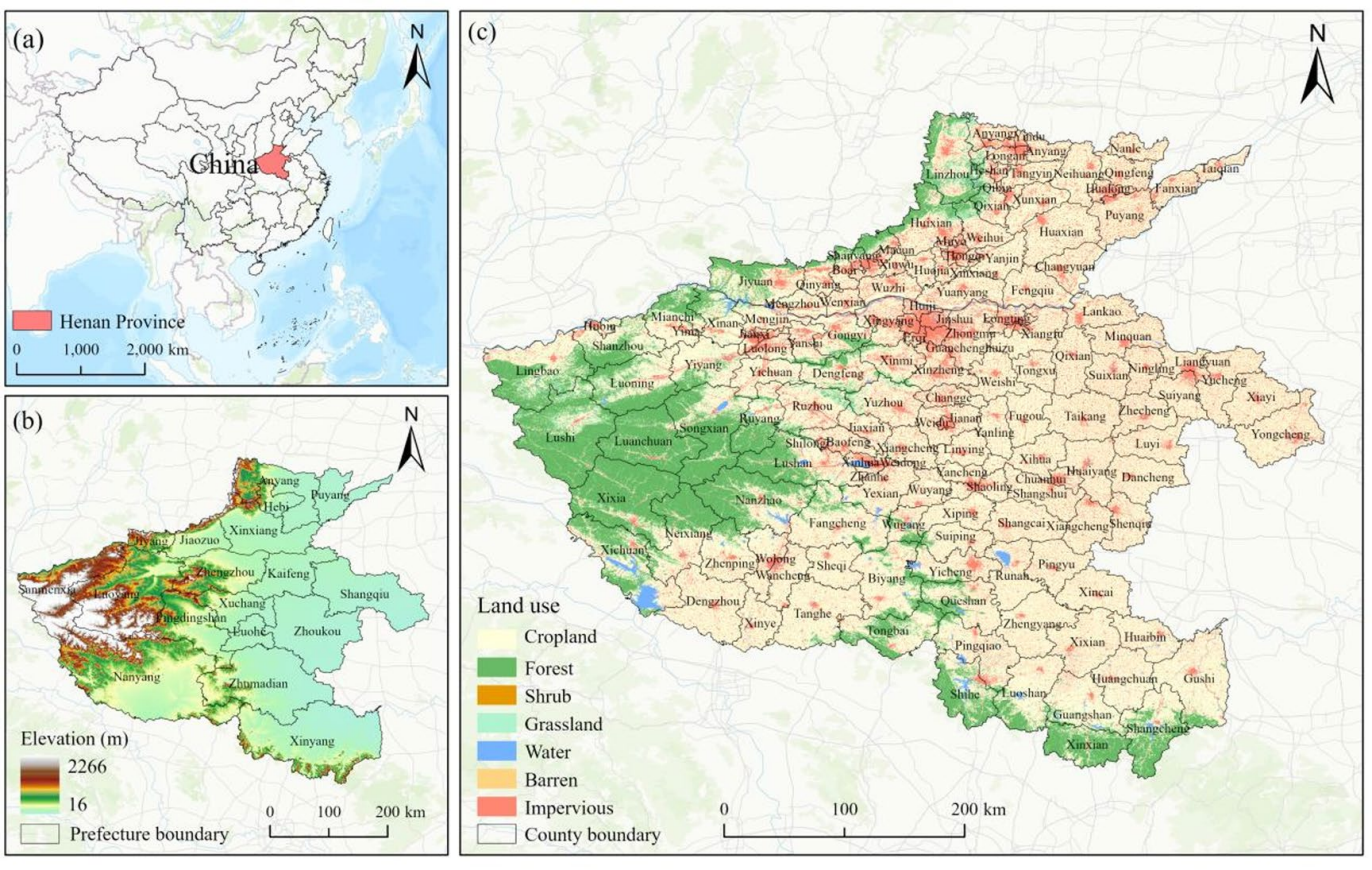
(**a**) Geographical location of Henan Province in China. (**b**) Topographical features and elevation (m) of Henan Province with prefecture boundaries. (**c**) Land use patterns and county-level administrative boundaries. Maps were generated using ArcGIS 10.8 (Environmental Systems Research Institute, Inc., Redlands, CA, USA; https://www.esri.com/en-us/arcgis/products/arcgis-desktop).

Located in central-eastern China within the middle and lower reaches of the Yellow River, Henan Province (110°21’-116°39’ E, 31°23’-36°22’ N) exhibits a distinct topographical gradient, characterized by higher elevations in the west and lower plains in the east (Fig. 1). While dominated by plains, the province also contains smaller areas of basins, mountains, and hills, covering a total area of approximately 167,000 km^2^.Henan has a continental monsoon climate, with rainfall concentrated between June and August and a frost-free period ranging from 201 to 285 days, providing an extended growing season favorable for crop cultivation. As one of China’s most important agricultural bases, the province produced 66.24 million tons of grain in 2023, accounting for approximately one-tenth of the national total. With a permanent population of 98.15 million and a GDP of 591.32 billion yuan (2023), Henan plays a pivotal role in national food security. Its significant grain output, combined with its large population and economic scale, underscores its strategic importance in China’s agricultural landscape and justifies its selection as a critical case study for analyzing cultivated land use dynamics.

### Data sources

The data used in this study were obtained from multiple authoritative sources to ensure accuracy and consistency. Socioeconomic data—including cereal crop planting area, total crop planting area, GDP, the proportion of the primary industry, urbanization rate, and population density from 2012 to 2022—were collected from the Henan Statistical Yearbook (2013–2023), county-level statistical yearbooks, and official reports on national economic and social development. In this study, the non-grain conversion rate was primarily calculated from county-level statistical data; meanwhile, to analyze the spatiotemporal variation of non-grain conversion and its driving factors and interactions, the above socioeconomic variables were interpolated using empirical Bayesian kriging to generate raster datasets with a resolution of 1 km^22,23^. Climate variables, including annual average temperature and annual precipitation, were acquired from the National Earth System Science Data Center (http://www.geodata.cn/) at a 1 km resolution. Elevation data were derived from the 30-m SRTM dataset (http://dwtkns.com/srtm30m/), while soil type data were obtained from the Resource and Environmental Science Data Center (https://www.resdc.cn/) at 1 km resolution. The multi-source datasets were uniformly resampled and aligned to facilitate integrated spatiotemporal analysis. The selection of data sources and spatial processing methods ensures high granularity and consistency across variables, providing a reliable foundation for analyzing the spatiotemporal patterns and drivers of non-grain conversion in Henan Province.

### Construction of index system

Based on existing literature and theoretical frameworks, this study selects driving factors of cultivated land non-grain conversion from two conceptual dimensions: natural-human interaction and spatiotemporal dynamics. The factors are organized into three categories: natural factors, human factors, and spatiotemporal composite factors. As summarized in Table 1, both natural and human factors are further decomposed into temporal variation and spatial differentiation components. These are then systematically integrated to derive spatiotemporal variation factors. After classifying each variable using the natural breaks method, homogeneous sampling points were established as initial data inputs. Through structured data cleaning procedures, including the removal of duplicate samples, the final analytical dataset consists of one dependent variable and 21 independent variables, totaling 18,037 valid samples. This curated dataset was subsequently analyzed using the geographical detector model to quantify driving mechanisms and interaction effects.

**Table 1:** Indicator selection.

Category			Variable name	Symbol
Dependent Variable		Rate of change over space and time	Spatiotemporal variation rate of non-grain cultivated land	En_Ng
Independent variable	Natural factor	Spatial variability factor	Altitude	DEM
			Elevation	Slope
			Soil type	Soil
			Spatial differentiation factor of annual average temperature	S_Tmp
			Spatial differentiation factor of annual precipitation	S_Pre
		Time-varying factors	Temporal variation factor of annual average temperature	H_Tmp
			Temporal variation factor of annual precipitation	H_Pre
	Humanistic factor	Spatial variability factor	Spatial differentiation factor of GDP	S_GDP
			Spatial differentiation factor of primary industry proportion	S_Agr
			Spatial differentiation factor of urbanization rate	S_Urb
			Spatial differentiation factor of population density	S_Den
		Time-varying factors	Temporal variation factor of GDP	H_GDP
			Temporal variation factor of primary industry proportion	H_Agr
			Temporal variation factor of urbanization rate	H_Urb
			Temporal variation factor of population density	H_Den
	Spatio-temporal dynamic factor	Spatial and temporal variability factors	Spatiotemporal variation factor of annual average temperature	En_Tmp
			Spatiotemporal variation factor of annual precipitation	En_Pre
			Spatiotemporal variation factor of GDP	En_GDP
			Spatiotemporal variation factor of primary industry proportion	En_Agr
			Spatiotemporal variation factor of urbanization rate	En_Urb
			Spatiotemporal variation factor of population density	En_Den

### Research methods

#### Non-grain conversion rate model

Drawing on existing research and guided by “Opinions on Preventing Farmland from Non-Grain Crops and Ensuring Stable Grain Production”, this study categorizes grain crops into three major types: wheat, corn, and rice, while non-grain crops mainly include vegetables, oil crops such as peanut, and cash crops such as cotton, and do not include fruit and nut orchards or land used for livestock rearing. The non-grain cropping rate is measured as the ratio of non-grain crop planting area to total crop planting area. This definition ensures data availability while avoiding calculation errors caused by duplicate statistical records.

1 $$\mathrm{R}=\left(1-\frac{\mathrm{F}}{\mathrm{G}}\right)\times 100\mathrm{\%}$$

where *R* is the non-grain cropping rate, *F* is the planting area of grain crops, and *G* is the total planting area of all crops.

#### Spatiotemporal variation model

This study analyzes the spatio-temporal variability of farmland non-grain cropping by building on the work of Zhao et al.^24^, Deng et al.^25^, and Zhang et al.^26^, and employs an improved spatio-temporal variability model to examine spatial variability, temporal variability, and joint spatio-temporal variability.

The spatial variability model incorporates the concept of spatial gradient to describe the spatial variation of geographical elements. In a homogeneous space, the spatial gradient of a geographic element is zero; however, in heterogeneous spaces, the gradient increases with the magnitude of directional changes in the element. This approach reflects the spatial variability, transition, and dynamic changes of geographic elements to a certain extent. In this study, the spatial gradient coefficient is used as the spatial variability index for the analyzed elements. The formula is as follows:

2 $${En}^{\mathrm{S}}=\frac{180}{\uppi }\times \mathrm{a}\mathrm{r}\mathrm{c}\mathrm{t}\mathrm{a}\mathrm{n}\sqrt{{\left(\frac{dz}{dx}\right)}^{2}+{\left(\frac{dz}{dy}\right)}^{2}}$$

3 $$\left(\frac{dz}{dx},\frac{dz}{dy}\right)=\frac{8}{{x}_{cs}}\left(\frac{4}{{wt}_{1}}\sum_{i=1}^{n}{cell}_{i}-\frac{4}{{wt}_{2}}\sum_{j=1}^{n}{cell}_{j}\right)$$

where ${En}^{\mathrm{S}}$ is the spatial variability of the element, $\frac{dz}{dx}$ and $\frac{dz}{dy}$ are the rates of change in the *x* and *y* directions of the pixels in the first-order neighborhood, ${x}_{cs}$ is the size of the pixel, ${wt}_{1}$ and ${wt}_{2}$ is the horizontal weighted counts of the effective pixels in the x and y directions, ${cell}_{i}$ and ${cell}_{j}$ is the value of the pixels in the *x* and* y* directions in the first-order neighborhood.

The temporal variation analysis employs information entropy metrics to quantify evolutionary patterns of system components. Theoretical foundations suggest observed residuals exhibit proportional relationships with stochasticity and heterogeneity, while inversely correlating with occurrence probabilities. The computational framework is expressed as:

4 $${En}^{H}=-\sum_{t=1}^{n}{P}_{t}\times ln{P}_{t}$$

5 $${P}_{t}=\frac{{W}_{t}^{-1}}{\sum_{t=1}^{n}{W}_{t}^{-1}} ,\sum {P}_{t}=1$$

6 $${W}_{t}=\frac{{\left({C}_{t}-{R}_{t}\right)}^{2}}{\sum_{t=1}^{n}{\left({C}_{t}-{R}_{t}\right)}^{2}} ,\sum {W}_{t}=1$$

where *En*^*H*^ is the elemental temporal variability in Nat, *P*_*t*_ is the elemental occurrence probability corresponding to time *t*, *W*_*t*_ is the sample residual percentage corresponding to time* t*, *C*_*t*_ is the elemental likelihood value corresponding to time *t*, and *R*_*t*_ is the elemental regression value corresponding to time *t*.

The composite spatiotemporal variability metric is derived through the mathematical synthesis of spatial variation coefficients and temporal variation indices, as formalized in the following equation:

7 $$\mathrm{E}\mathrm{n}=\frac{1}{2}\left\{\left[\frac{{En}^{\mathrm{S}}}{\mathrm{m}\mathrm{a}\mathrm{x}\left({En}^{\mathrm{S}}\right)}\right]+\left[\frac{{En}^{H}}{\mathrm{m}\mathrm{a}\mathrm{x}\left({En}^{H}\right)}\right]\right\}$$

where *En* is the composite spatiotemporal variation coefficient of geographical elements.

#### Spatial autocorrelation model

The spatial autocorrelation analytical framework, comprising global and local measurement dimensions, effectively evaluates spatial clustering patterns of geographical phenomena^27^. Global Moran’s I quantifies overall spatial autocorrelation intensity of agricultural land-use transitions, which can be expressed by Eq. (8). Local Moran’s I represents the local spatial autocorrelation, which is used to judge the distribution and clustering characteristics of the phenomenon of non-grain in the local space and can be expressed by Eq. (9).

8 $${I}_{g}=\frac{n\sum_{i=1}^{n}\sum_{j=1}^{n}{W}_{ij}\left({x}_{i}-\overline{x }\right)\left({x}_{j}-\overline{x }\right)}{\sum_{i=1}^{n}\sum_{j=1}^{n}{W}_{ij}\sum_{i=1}^{n}{\left({x}_{i}-\overline{x }\right)}^{2}}$$

9 $${I}_{l}=\frac{\left({x}_{i}-\overline{x }\right)\sum_{j=1}^{n}{W}_{ij}\left({x}_{i}-\overline{x }\right)}{\frac{1}{n}\sum_{i-1}^{n}{\left({x}_{i}-\overline{x }\right)}^{2}}$$

where, n is the total spatial units; ${\mathrm{x}}_{\mathrm{i}}$ and ${\mathrm{x}}_{\mathrm{j}}$ are the observed values of units i and j, respectively; $\overline{\mathrm{x} }$ is the mean value across all units; and ${\mathrm{W}}_{\mathrm{i}\mathrm{j}}$ is the spatial weight matrix, cells i and j are weighted 1 if they are adjacent and 0 if they are not.

#### Geodetector model

The geodetector analytical framework, pioneered by Wang et al.^28^ enables systematic identification of spatial heterogeneity patterns and their underlying determinants through quantitative spatial modeling. This framework comprises four core analytical modules: factor contribution assessment, interaction effect evaluation, ecological association analysis, and risk pattern identification. The current study specifically focuses on the first two components. The factor analysis module quantifies both spatial distribution characteristics and determinant influence magnitudes through the following computational mechanism:

10 $$\mathrm{q}=1-\frac{\sum_{\mathrm{h}=1}^{\mathrm{L}}{\mathrm{N}}_{\mathrm{h}}{\upsigma}_{\mathrm{h}}^{2}}{\mathrm{N}{\upsigma }^{2}},\mathrm{h}=\mathrm{1,2},...,\mathrm{L}$$

where *q* is the explanatory power of factor *X* to *Y*, *q* ∈ [0,1], the larger the value the stronger the explanatory power to *Y*, *h* = 1,2,……, *L* is the stratification of *X* or *Y*, ${N}_{h}$ is the number of units in stratum *h*, *N* is the number of units in the whole region, ${\sigma}_{h}^{2}$ is the variance of the *Y* value corresponding to stratum *h*, and *σ*^*2*^ is the variance of the *Y* value in the whole region.

The interaction analysis module effectively identifies synergistic relationships between driving factors, with specific interaction patterns categorized in Table 2.

**Table 2:** Type of interaction between different factors.

Criterion	Interaction type
q(X_1_ ∩ X_2_) < Min(q(X_1_) ,q(X_2_))	Nonlinear weakening
Min(q(X_1_),q(X_2_)) < q(X_1_ ∩ X_2_) < Max(q(X_1_),q( X_2_))	Single-factor nonlinear weakening
q(X_1_ ∩ X_2_) > Max(q(X_1_),q( X_2_))	Bifactor enhancement
q(X_1_ ∩ X_2_) = q(X_1_) + q(X_2_)	Independent
q(X_1_ ∩ X_2_) > q(X_1_) + q(X_2_)	Nonlinear enhancement

## Results

### Temporal variation characteristics analysis

**Fig. 2:**
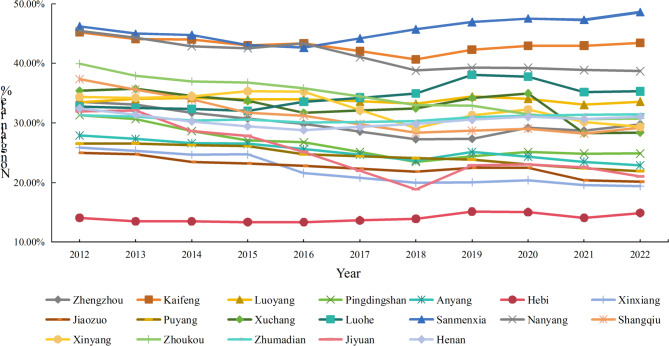
Temporal changes in cultivated land non-grain conversion rates across prefecture-level cities in Henan Province (2012–2022).

From 2012 to 2022, the non-grain conversion rate of cultivated land in Henan Province exhibited a distinct U-shaped trend, characterized by an initial decline followed by a subsequent rebound, with fluctuations consistently occurring around 30% throughout the period (Fig. 2). Despite being one of China’s major grain-producing regions, Henan maintains a relatively high level of non-grain conversion. A more detailed examination reveals that the conversion rate decreased annually from 32.5% to 28.8% between 2012 and 2016, representing a decline of 3.64 percentage points. This trend reversed after 2016, with the rate steadily increasing to 31.1% by 2022—a rise of 2.24 percentage points. The year 2016 thus represents a critical turning point in the evolution of non-grain conversion in the province. This reversal aligns with shifting dietary patterns, as consumer preferences progressively transitioned from staple grains toward diversified products including meat, dairy, and horticultural crops. Concurrently, the Henan Agricultural and Rural Economic Development Plan (2016–2020) explicitly promoted structural adjustments toward higher-value crops such as peanuts, livestock, and fruits. These policy-driven agricultural transformations facilitated the sustained expansion of non-grain cultivation, culminating in the highest conversion rate in seven years by December 2022.

At the prefectural level, cities such as Kaifeng, Luoyang, Sanmenxia, and Nanyang demonstrated conversion rates exceeding the provincial average. Kaifeng, in the eastern alluvial plain, is a major base for vegetables and peanuts, whose expansion has replaced part of the wheat–maize area and kept its non-grain rate relatively high. Nanyang, as a typical “grain–cotton–oil–tobacco” region, has seen cotton, oil crops and tobacco reduce the share of grain crops and keep the non-grain rate above the provincial average. Xinxiang combines high-standard grain bases with seed breeding and greenhouse vegetables, forming a “grain + economic crops” pattern and a modest rise in the non-grain rate, while in Jiyuan, with its small cultivated land base, even limited expansion of high-value crops can noticeably change the non-grain rate. In mountainous Sanmenxia, the shift from grain to edible fungi, medicinal plants, fruit trees and livestock uses on part of the cultivated land has led to a persistently high non-grain rate.

At the county level, significant spatial heterogeneity characterized the non-grain conversion rate across Henan Province from 2012 to 2022. Distinct regional patterns emerged, with notable increases observed in Fengquan and Muye Districts (Xinxiang), Qibin District (Hebi), Guancheng Hui and Huiji Districts (Zhengzhou), Lushi County (Sanmenxia), and Chanhe Hui District (Luoyang). Among these, Qibin District recorded the most substantial increase of 36.03%, followed by Chanhe Hui District with a rise of 23.29%.Conversely, several areas exhibited marked declines exceeding 10%, including Heshan District (Hebi), Beiguan and Wenfeng Districts (Anyang), Luolong District (Luoyang), and Wuzhi County (Jiaozuo). Luolong District demonstrated the most pronounced decrease at 26.40%. In contrast, counties such as Xinzheng and Zhongmu (Zhengzhou), Ruyang (Luoyang), and Zhenping and Wolong (Nanyang) maintained relative stability, with fluctuation margins below 2%. Notably, Ruyang County exhibited minimal variation, registering a change of only 0.08% throughout the study period.

**Fig. 3:**
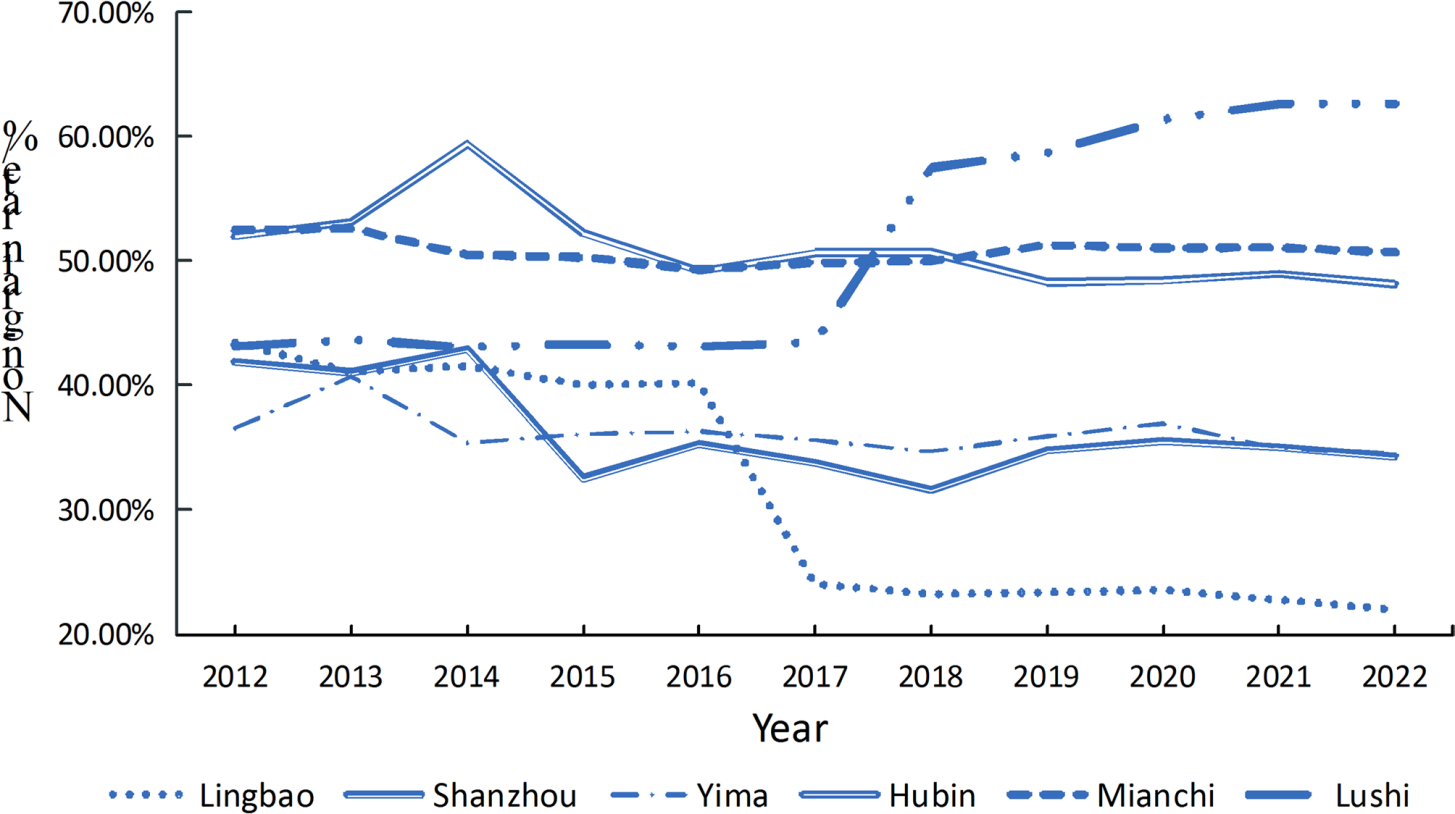
Temporal changes in cultivated land non-grain conversion rates in Sanmenxia City (2012–2022).

Given the large number of county-level units in Henan Province, Sanmenxia City—which exhibited the highest overall non-grain conversion rate—was selected for detailed case analysis. Within Sanmenxia, distinct temporal patterns emerged at the county scale between 2012 and 2022 (Fig. 3). Lingbao City maintained relatively stable conversion rates from 2012 to 2016, followed by a sharp decline of 16.12% in 2017 and subsequent gradual decreases, which is partly related to the adjustment of low-efficiency fruit-oriented plots and the restoration of grain cultivation on some piedmont plains and terraced fields. Shanzhou District recorded a more moderate reduction of 7.52%, while Hubin District showed rapid growth during 2012–2014 before entering a declining phase. In contrast, Lushi County demonstrated a continuous upward trend, peaking at 62.58% in 2022—the highest level observed in Sanmenxia, as the continuous expansion of edible fungi, medicinal plants and under-forest production onto previously grain-dominated plots has squeezed the sown area of grain crops and contributed to its persistently high and rising non-grain rate. Yima City and Mianchi County maintained comparatively stable conversion rates throughout the study period. The pronounced non-grain conversion in Sanmenxia can be attributed to its distinctive topographic and agricultural conditions. Characterized predominantly by mountains, hills, and loess plateaus, the region faces inherent constraints for large-scale grain production. However, these same conditions have facilitated the development of specialized agricultural systems, including fruit and nut orchards, livestock rearing, edible fungi cultivation, vegetable production, and tobacco farming. These activities are further supported by integrated agroforestry models that combine vegetable and mushroom cultivation with livestock grazing under fruit trees, creating economically viable alternatives to traditional grain cropping.

### Spatial differentiation characteristics analysis

#### Overall spatial distribution characteristics

Significant disparities in non-grain conversion levels were observed among the 157 county-level units in Henan Province from 2012 to 2022. To facilitate systematic comparison across the 11-year study period, data were stratified using the natural breaks classification method. Three representative temporal cross-sections—2012, 2017, and 2022—were selected for spatial visualization through ArcGIS 10.8, effectively revealing the spatiotemporal evolution of non-grain conversion patterns across the province. Based on their conversion rates, the county-level units were classified into five distinct zones: heavier non-grain (> 53.44%), heavy non-grain (39.02%–53.44%), medium non-grain (28.80%–39.02%), light non-grain (19.19%–28.80%), and lighter non-grain (< 19.19%). It should be noted that Zhongyuan District of Zhengzhou City was excluded from the analysis from 2018 onward, as it no longer contained agricultural land and consequently exhibited no cultivated land non-grain conversion.

**Fig. 4:**
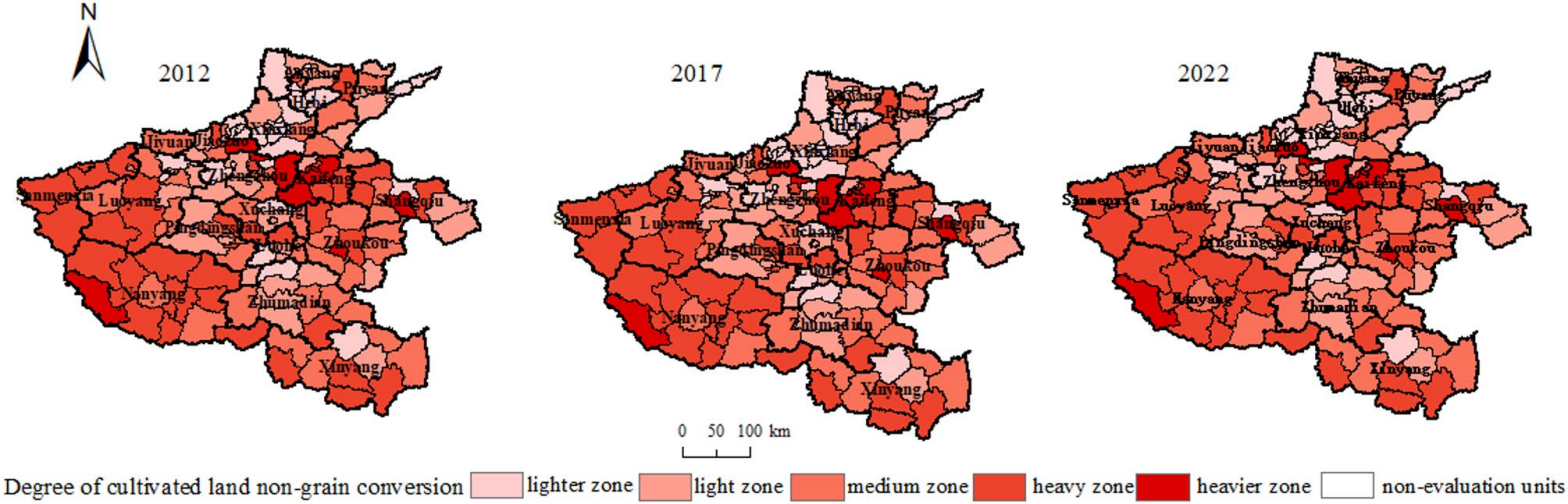
Spatial distribution of cultivated land non-grain conversion intensity in Henan Province (2012–2022). Maps were generated using ArcGIS 10.8 (Environmental Systems Research Institute, Inc., Redlands, CA, USA; https://www.esri.com/en-us/arcgis/products/arcgis-desktop).

Analysis of the spatial distribution of non-grain conversion rates in Henan Province reveals distinct geographical patterns across the three reference years (2012, 2017, and 2022; Fig. 4). Throughout the study period, the western, southern, and selected central regions consistently exhibited higher conversion rates, while northern areas maintained comparatively lower levels. In 2012, critical conversion zones were identified in Zhongmu County (Zhengzhou), Weishi County and Xiangfu District (Kaifeng), Wuzhi County (Jiaozuo), Xichuan County (Nanyang), and Suiyang District (Shangqiu). Areas with minimal conversion were predominantly concentrated in northern counties of Anyang, Hebi, and Xinxiang, while moderate conversion zones displayed a scattered distribution across the province.By 2017, while the overall spatial pattern remained relatively stable, several notable transitions occurred: Xiangfu District shifted from the critical to the severe category; Weishi County and Suiyang District were downgraded to moderate zones; Yongcheng City escalated from mild to critical status; and Nanzhao County and Shihe District entered the critical classification. In 2022, critical zones further expanded to include Xichuan County, Nanzhao County (Nanyang), Lushi County (Sanmenxia), Shihe District (Xinyang), and Zhongmu County (Zhengzhou). Northern regions continued to demonstrate persistently lower conversion intensities, maintaining the fundamental north–south differentiation observed throughout the study period.

#### Spatial correlation analysis

**Fig. 5:**
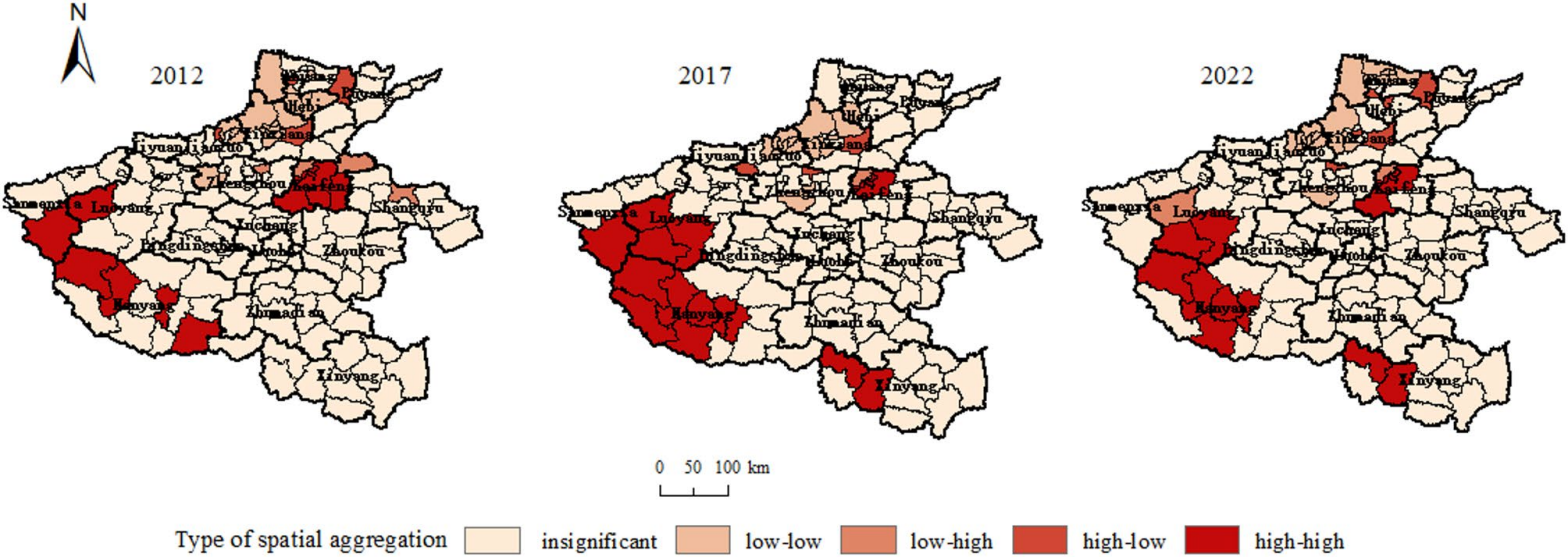
Spatial aggregation types of cultivated land non-grain conversion in Henan Province (2012–2022). Maps were generated using ArcGIS 10.8 (Environmental Systems Research Institute, Inc., Redlands, CA, USA; https://www.esri.com/en-us/arcgis/products/arcgis-desktop).

The Global Moran’s I indices for 2012, 2017, and 2022 all demonstrated statistically significant positive values (Z > 2.58, *p* < 0.01), indicating pronounced spatial clustering of non-grain conversion rates across Henan Province. Local spatial autocorrelation analysis (Fig. 5) revealed that high-high and low-low clusters represented the dominant spatial patterns, with other cluster types exhibiting limited spatial representation. In 2012, 13 county-level units formed high-high clusters, primarily concentrated in Kaifeng, Sanmenxia, Luoyang, and Nanyang, while 16 low-low clusters were identified in Anyang, Hebi, Xinxiang, Jiaozuo, and Zhengzhou. By 2017, the number of high-high clusters showed a net increase of four counties compared to 2012. During this period, Kaifeng City experienced a reduction in high-high clustering units, while Luoyang, Nanyang, and Xinyang Cities saw notable expansions of such clusters. Concurrently, low-low clusters decreased by two counties, with Wenfeng District and Linzhou City (Anyang) transitioning from significant low-low clustering to non-significant spatial patterns. In 2022, the number of high-high clusters declined to 14, primarily concentrated in Luoyang, Nanyang, Xinyang, and Kaifeng Cities. Low-low clusters remained predominantly distributed across northern Henan, particularly within Anyang, Xinxiang, and Jiaozuo Cities, maintaining the persistent north–south spatial differentiation observed throughout the study period.

#### Spatiotemporal variation characteristics analysis

**Fig. 6:**
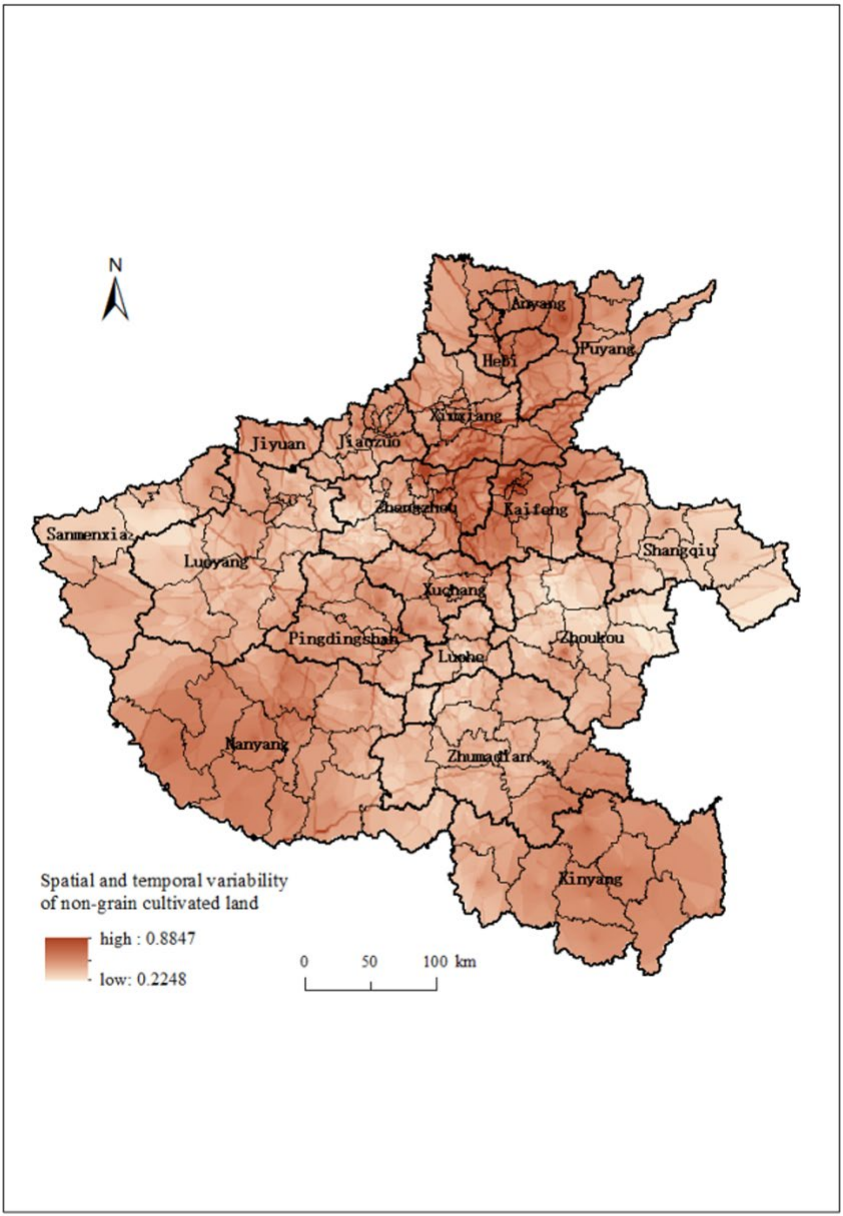
Spatiotemporal variation rate of cultivated land non-grain conversion in Henan Province (2012–2022). Maps were generated using ArcGIS 10.8 (Environmental Systems Research Institute, Inc., Redlands, CA, USA; https://www.esri.com/en-us/arcgis/products/arcgis-desktop).

Figure 6 illustrates the spatiotemporal variation patterns of cultivated land non-grain conversion across Henan Province during the 2012–2022 period. Areas exhibiting high spatiotemporal variation rates are predominantly concentrated in northern Henan, with secondary clusters identified in southwestern and northeastern regions. Regional analysis reveals peak variation values (maximum: 0.8847) in the central-northern zone, encompassing Huiji District, Jinshui District, and Zhongmu County (Zhengzhou); Yuanyang County and Changyuan City (Xinxiang); as well as Longting District and Xiangfu District (Kaifeng). These areas represent regions undergoing intensive spatiotemporal transitions in non-grain conversion. Conversely, low-variation zones (minimum: 0.2248) are clustered in southern Yongcheng City (Shangqiu); southwestern Luyi County and northeastern Dancheng County (Zhoukou); and southern Shanzhou District (Sanmenxia). These regions demonstrate relatively stable transition patterns throughout the study period, forming distinct spatial counterparts to the high-variation areas.

Analysis at the municipal level reveals distinct spatial patterns in spatiotemporal variation rates, with elevated values observed in northeastern Zhengzhou, northwestern Kaifeng, southern Xinxiang, eastern Anyang, central Nanyang, and central Xinyang. These areas contrast sharply with regions exhibiting lower variation rates, particularly Zhoukou, Shangqiu, and Sanmenxia. This pronounced spatial heterogeneity primarily stems from fundamental disparities in natural endowments and economic development levels. Regions characterized by favorable agro-ecological conditions and robust economic foundations demonstrate enhanced capacity for implementing grain security policies, thereby maintaining greater stability in cultivated land use. Conversely, areas constrained by environmental limitations and insufficient agricultural investment face reduced incentives for grain cultivation, creating dual pressures of yield constraints and accelerated non-grain conversion processes. The interaction between socioeconomic capacity and biophysical constraints thus creates a feedback mechanism that reinforces regional divergence in non-grain conversion trajectories across the province.

### Driving forces of cultivated land non-grain conversion dynamics

#### Driving factor analysis of cultivated land non-grain conversion

The explanatory power of various factors regarding the spatiotemporal variation of non-grain conversion was quantified using the geographical detector model (Table 3). In terms of temporal versus spatial dimensions, temporal change factors exhibited a mean explanatory power (q-value) of 0.0516, exceeding that of spatial differentiation factors by 0.0260. This indicates that temporal dynamics exert a stronger influence on non-grain conversion than spatial heterogeneity, with more pronounced variation trends over time. From the perspective of factor categories, socioeconomic factors demonstrated a higher mean q-value (0.0540) compared to natural factors (0.0155), revealing that socioeconomic drivers play a dominant role in shaping the spatiotemporal patterns of non-grain conversion across Henan Province. Furthermore, with the exception of the proportion of primary industry and urbanization rate, the q-values of spatiotemporal variation factors for all other elements surpassed those of their respective spatial differentiation and temporal change components. This suggests that the interaction between temporal and spatial dimensions amplifies the driving effects of individual factors, highlighting the importance of integrated spatiotemporal analysis in understanding land use transitions.

**Table 3:** Explanatory power of driving factors on spatiotemporal variation of cultivated land non-grain conversion.

Factor		q-value
Natural factor	DEM	0.0220
	Slope	0.0026
	Soil	0.0523
	S_Tmp	0.0069
	S_Pre	0.0024
	H_Tmp	0.0049
	H_Pre	0.0174
Humanistic factor	S_GDP	0.0713
	S_Agr	0.0370
	S_Urb	0.0301
	S_Den	0.0058
	H_GDP	0.0892
	H_Agr	0.0114
	H_Urb	0.0453
	H_Den	0.1416
Spatial and temporal variability factors	En_Tmp	0.0179
	En_Pre	0.0408
	En_GDP	0.1139
	En_Agr	0.0093
	En_Urb	0.0421
	En_Den	0.1519

Among all factors examined, population density and GDP demonstrated the strongest influence on the spatiotemporal variation of non-grain conversion. Specifically, the spatiotemporal variation factor of population density (En_Den) exhibited the highest explanatory power (q = 0.1519), accounting for 15.19% of the observed variation, followed by its temporal variation factor (H_Den, q = 0.1416), explaining 14.16% of the changes. This indicates that both spatiotemporal dynamics and temporal trends of population density serve as significant drivers of non-grain conversion. The underlying mechanism involves the substantial pressure exerted by high population density, where increased demographic concentration intensifies competition for limited land resources. To meet escalating demands for food and livelihoods, farmers are incentivized to transition toward higher-value cash crops, thereby accelerating cultivated land conversion. Furthermore, population density correlates strongly with land-use intensification and industrial restructuring. In densely populated regions, scarce land availability promotes more intensive utilization patterns, while concurrent economic development drives a relative decline in the agricultural sector’s share alongside expansion of industrial and service activities. Collectively, these processes redirect cultivated land toward non-grain production. GDP variations also showed substantial explanatory power across dimensions: the spatiotemporal variation factor (En_GDP, q = 0.1139), temporal factor (H_GDP, q = 0.0892), and spatial factor (S_GDP, q = 0.0713). This confirms GDP’s consistent influence regardless of analytical dimension, with spatiotemporal interactions enhancing its explanatory capacity beyond isolated temporal or spatial effects. In contrast, environmental factors including precipitation spatial variation (S_Pre, q = 0.0024) and slope gradient (Slope, q = 0.0026) demonstrated negligible explanatory power, indicating their limited statistical significance in driving non-grain conversion patterns within the study context.

#### Interactive factor analysis of cultivated land non-grain conversion

Interaction detection analysis (Table 4) reveals that all factor combinations exhibit either bilinear or nonlinear enhancement patterns, indicating that non-grain conversion of cultivated land stems from synergistic effects between natural and socioeconomic drivers rather than isolated determinants. All interaction q-values exceeded the explanatory power of individual factors, confirming that cross-factor synergies critically amplify the conversion process.

**Table 4:** Results of interactive factor analysis.

	DEM	Slope	Soil	En_Tmp	En_Pre	En_GDP	En_Agr	En_Urb	En_Den
DEM	0.0220								
Slope	0.0295	0.0026							
Soil	0.0948	0.0600	0.0523						
En_Tmp	0.0520	0.0259	0.0904	0.0179					
En_Pre	0.1243	0.0660	0.1383	0.0736	0.0408				
En_GDP	0.1712	0.1227	0.1908	0.1392	0.1648	0.1139			
En_Agr	0.0326	0.0169	0.0851	0.0487	0.0752	0.1373	0.0093		
En_Urb	0.1555	0.0877	0.2093	0.1013	0.0878	0.1334	0.0721	0.0421	
En_Den	0.1907	0.1616	0.1898	0.1914	0.2075	0.2781	0.2183	0.1980	0.1519

Interaction effect analysis identified the strongest synergistic interaction between the spatiotemporal variation factors of population density and GDP (q = 0.2781), jointly explaining 27.81% of the conversion dynamics. This indicates a significant coupling effect between demographic and economic drivers. Regions experiencing simultaneous growth in both population density and economic output exhibited amplified non-grain conversion, a pattern attributable to several interrelated mechanisms. Enhanced transportation infrastructure, rising consumption levels, and diversified agricultural demand—all characteristic of advanced socioeconomic development—collectively drive this trend. Improved regional accessibility and higher living standards create economic incentives for agricultural producers to shift toward high-value cash crops, thereby optimizing land-use profitability. Meanwhile, constrained natural resources and growing consumption demands further accelerate the reallocation of production factors toward non-grain sectors. These processes form synergistic feedback loops that intensify the spatiotemporal dynamics of cultivated land conversion.

From both natural and socioeconomic dimensions, the interaction q-values among socioeconomic factors consistently surpass those of natural factor interactions and nature-socioeconomic cross-interactions. Furthermore, the combined influence of interacting socioeconomic factors exceeds the explanatory power of any single element. These findings demonstrate that socioeconomic drivers play a predominant role in shaping the spatiotemporal evolution of cultivated land non-grain conversion, while natural factors primarily function to amplify the effects of socioeconomic drivers. This hierarchical relationship confirms the dominance of socioeconomic interactions in governing conversion patterns, with natural factors assuming a secondary, yet catalytic, role in the process.

## Conclusions and policy implications

### Conclusions

1. The non-grain conversion rate of cultivated land in Henan Province followed a distinct U-shaped trajectory from 2012 to 2022, characterized by an initial decline followed by a marked resurgence, with considerable variation observed across municipal units. At the county level, heterogeneous spatial patterns emerged: Fengquan District, Muye District (Xinxiang), and Qibin District (Hebi) experienced substantial increases in conversion rates, whereas Heshan District (Hebi), Beiguan District, and Wenfeng District (Anyang) registered declines exceeding 10% relative to 2012. In contrast, counties including Xinzheng, Zhongmu (Zhengzhou), and Ruyang (Luoyang) maintained relative stability with minimal fluctuation. Spatially, elevated conversion intensities consistently clustered in western, southern, and select central regions, forming a clear contrast with persistently lower rates observed in northern Henan. This period also witnessed a discernible expansion of high-intensity zones. Global and local spatial autocorrelation analyses confirmed significant clustering effects, dominated by high-high and low-low aggregation patterns, while high-low and low–high outlier clusters remained spatially limited. Spatiotemporal variability was most pronounced in northern, southwestern, and northeastern regions, reflecting differentiated regional trajectories of land-use transition.
2. Factor detection results indicate that temporal variation factors possess significantly greater explanatory power (mean q-value difference: + 0.0260) than spatial differentiation factors, demonstrating that temporal dynamics outweigh spatial heterogeneity in driving non-grain conversion patterns. Socioeconomic factors consistently dominate natural factors in explaining the spatiotemporal variability of cultivated land conversion across Henan Province. Population density and GDP emerged as the most influential determinants. Specifically, the spatiotemporal variation factor of population density (En_Den) achieved the highest explanatory power (q = 0.1519), followed by its temporal variation component (H_Den, q = 0.1416). The spatiotemporal, temporal, and spatial dimensions of GDP (En_GDP, H_GDP, S_GDP) also showed substantial effects. In contrast, environmental variables including precipitation spatial variation (S_Pre, q = 0.0024) and slope gradient (Slope, q = 0.0026) demonstrated negligible explanatory capacity and statistical significance.
3. Interaction detection results reveal that all factor combinations exhibit either bilinear or nonlinear enhancement effects, with nonlinear interactions representing the predominant pattern. Comparative analysis of interaction q-values identified the strongest synergistic effect between the spatiotemporal variation factors of population density and GDP (q = 0.2781), confirming their coupled dominance in driving the spatiotemporal evolution of non-grain conversion. From a typological perspective, interactions among socioeconomic factors demonstrate substantially greater explanatory power than those involving natural factors or natural-socioeconomic cross-interactions. This hierarchical structure underscores the primacy of socioeconomic drivers in governing conversion patterns, while natural factors play a secondary—though catalytically significant—role by amplifying anthropogenic effects.
4. The spatiotemporal evolution of non-grain cultivated land in Henan Province stems from multi-dimensional interactions between natural and anthropogenic factors across temporal and spatial scales. While natural factors generally exhibit weaker direct influence than socioeconomic drivers, specific biophysical conditions—such as the mountainous and hilly terrain in Sanmenxia that constrains large-scale grain production—create favorable environments for specialized non-grain sectors including forestry and horticulture. Among anthropogenic drivers, population density emerges as the primary determinant. High population concentrations intensify competition for land resources, prompting farmers to shift toward higher-value non-grain crops to enhance livelihood security. This demographic pressure simultaneously accelerates land-use intensification and industrial restructuring, further reinforcing conversion trends. Economic development, measured through GDP, constitutes another major driver: economically advanced regions benefit from enhanced transportation infrastructure, diversified consumption demand, and stronger market incentives for profit-oriented agricultural transitions. Temporally dynamic factors demonstrate stronger explanatory power than spatial heterogeneity, yet their synergistic interactions produce the most substantial effects. The coupled interaction between population density and GDP variations (q = 0.2781) exemplifies how socioeconomic drivers operate in concert to accelerate conversion processes. This multi-layered interplay between temporal dynamics, spatial differentiations, and factor interactions ultimately shapes the complex and persistent patterns of cultivated land conversion observed across the province.

### Policy suggestion

Based on the empirical findings, this study proposes the following policy recommendations to support sustainable cultivated land use and grain security:

1. Strengthen the protection of grain cultivation areas and enhance comprehensive production capacity. Agricultural policy should adhere to the principle of "stabilizing acreage, optimizing quality, regulating utilization, and unleashing potential," establishing a hierarchical land-use system that prioritizes staple grain crops (rice, wheat, and corn) to ensure their sown area remains secure. Within this framework, non-grain land use should be rationally allocated to meet diversified agricultural demands, while market mechanisms should guide the adjustment of oversupplied cash crops to prevent disorderly expansion. Comprehensive land consolidation should be systematically implemented, incorporating the integrated development of agricultural land, rural construction areas, inefficient industrial and mining land, rehabilitated land, and undeveloped land. By merging fragmented plots into contiguous farmland, land-use efficiency can be significantly improved. For non-grain crops, priority should be given to the utilization of marginal lands, idle plots, or consolidated fragmented areas, thereby minimizing encroachment on core grain-producing zones.
2. Implement Dynamic Monitoring and Targeted Regulation in High-Risk Spatial Clusters. As evidenced by the spatial autocorrelation and spatiotemporal variation analyses, the non-grain conversion of cultivated land exhibits pronounced spatial heterogeneity, forming distinct high-high agglomerations (e.g., in Nanyang and Luoyang) alongside localized high-variation temporal hotspots. This spatial clustering necessitates a paradigm shift from uniform administrative measures toward spatially targeted resource allocation. Policymakers should establish a dynamic monitoring mechanism strictly prioritizing these high-risk spatiotemporal zones and regions undergoing rapid socioeconomic transitions. By implementing grid-based spatial management tailored to the identified high-high clusters, local authorities can deploy effective early warning systems, mitigate disorderly non-grain expansion, and optimize regulatory efficiency, thereby circumventing the limitations of a homogenous, province-wide regulatory framework.
3. Implement Terrain-Adaptive and Regionally-Differentiated Governance Strategies. Grain production policies should be tailored to local topographic conditions and development levels. Based on distinct geomorphological characteristics—including mountainous, hilly, and plain areas—planting structures should be optimized according to regional economic conditions, fiscal capacity, and farmers’ adaptability, with tiered implementation standards to balance development goals and practical feasibility. For instance, in western Henan—such as Sanmenxia and Nanyang, where mountainous and hilly terrain dominates and agricultural mechanization remains limited—targeted investments and policy support should encourage the development of specialty non-grain crops while ensuring baseline grain output. This approach aligns agricultural practices with local geographic constraints and opportunities. Conversely, in plain areas with superior cultivation conditions, grain production must be prioritized through stabilized planting acreage, enhanced production efficiency, and the construction of high-standard farmland. This differentiated strategy balances regional comparative advantages with national food security objectives, contributing to a resilient and sustainable agricultural system across diverse landscapes.
4. Prioritize Population Density Dynamics in Zoning Non-Grain Cultivation. Given the research-confirmed dominance of population density as the primary driver of cultivated land conversion, strategic coordination between grain production and demographic dynamics is essential. Regional authorities should systematically evaluate current and projected demands for both staple and non-staple crops, aligning cultivation zoning with national food security objectives and local resource endowments. This scientifically informed demarcation ensures agricultural diversification without compromising absolute grain security. Simultaneously, enhanced land-use supervision should prevent high-quality cropland from being encroached upon by construction projects and curb excessive non-grain expansion. At the national level, incentivized agricultural land transfer mechanisms are recommended to promote scaled operations, guiding farming households to prioritize grain production while permitting moderate non-grain cultivation where appropriate. This multi-tiered governance framework balances demographic pressures with agricultural sustainability, enabling systematic land allocation that responds to both developmental needs and ecological imperatives.
5. Build Multi-Stakeholder Synergy for Sustainable Grain Production. To ensure sustainable grain production, governmental bodies, farmers, and diverse social actors should establish collaborative alliances that integrate resources and align interests. While promoting economic growth and urbanization, relevant authorities should strengthen interdepartmental coordination under clear policy leadership. Key measures include implementing farmland rehabilitation programs, increasing subsidies for agricultural machinery, enhancing cultivation technical support, and improving grain production profitability to incentivize farmer engagement. Concurrently, cross-sector cooperation should advance scientific land-use planning for both enterprises and farming households, strengthen monitoring of cultivated land utilization, and rigorously penalize illegal encroachments on permanent basic farmland. Targeted subsidies should be deployed to modernize the grain industry, fostering efficient, ecological, and sustainable development models. Inspired by the governance philosophy of initiatives such as the "Thousand-Village Demonstration, Ten-Thousand-Village Improvement" project, farmer-centered approaches must be prioritized to protect their principal status and legitimate rights. This requires effective coordination among four core actors—government, market, rural collectives, and farmers—through a mechanism that combines state investment guidance, collective-farmer co-management, and broad societal participation, collectively reinforcing the foundation of national food security.

### Research prospect

This study systematically examined the spatiotemporal evolution and driving mechanisms of cultivated land non-grain conversion in Henan Province from 2012 to 2022 using spatiotemporal variation modeling, spatial autocorrelation analysis, and geographical detector modeling at the county scale. However, several methodological limitations should be acknowledged. The non-grain conversion rate was calculated solely based on the ratio of non-staple crop sown area to total agricultural sown area, and socioeconomic data were exclusively sourced from statistical yearbooks. Although this sown-area-based indicator reflects differences in multiple cropping to some extent, it still cannot distinguish whether non-grain conversion is driven by increased multiple cropping of non-grain crops or by changes in their actual sown area; future studies could refine this indicator by integrating higher-resolution, plot-level crop maps and detailed cropping-system data. In addition, Important micro-level drivers—such as farmer decision-making behaviors, agricultural technology adoption, and household-level socioeconomic factors—were not directly investigated. Future research should incorporate micro-scale surveys, behavioral experiments, or participatory approaches to elucidate the intrinsic relationships between land conversion patterns and agricultural stakeholders’ decision-making mechanisms. Interdisciplinary methodologies integrating agricultural economics, behavioral geography, and sociology could further unravel the complex behavioral drivers underlying non-grain conversion. Such advances would enable more precise identification of causal pathways and facilitate targeted policy interventions for cultivated land protection and sustainable agricultural transition.

## Data Availability

The datasets generated and analysed during the current study are not publicly available due to institutional data management policy restrictions, but are available from the corresponding author upon reasonable request.
